# Severity of Anemia among Children under 36 Months Old in Rural Western China

**DOI:** 10.1371/journal.pone.0062883

**Published:** 2013-04-23

**Authors:** Wenlong Gao, Hong Yan, DuolaoWang, Shaonong Dang, Leilei Pei

**Affiliations:** 1 Department of Epidemiology and Health Statistics, School of Public Health, College of Medicine, Xi'an Jiaotong University, Xi'an, Shaanxi, People's Republic of China; 2 Department of Medical Statistics, London School of Hygiene and Tropical Medicine, London, United Kingdom; Robert Wood Johnson Medical School, United States of America

## Abstract

**Objective:**

To describe severity of anemia and explore its determinants among children under 36 months old in rural western China.

**Study Design:**

The family information of 6711 children was collected and their hemoglobin was measured in 2005. A generalized estimated equation (GEE) linear model was used to identify the determinants of severity of childhood anemia.

**Results:**

The prevalence of mild, moderate and severe anemia among these children was 27.4%, 21.9% and 3.2% respectively. GEE model analysis showed that province-level region and severity of maternal anemia affected the severity of childhood anemia not only in 0–5 months but also beyond 5 months. In addition, children aged 0–5 months in families using iron pot (coefficient = −0.26 95%CI −0.41,−0.12) had seldom more severe anemia, and children aged 6–36 months in families more than 4 members (coefficient = −0.03 95%CI −0.06,−0.01) or of Han ethnicity (coefficient = −0.08 95%CI −0.13,−0.04) seldom had more severe anemia but boys (coefficient = 0.03 95%CI 0.01,0.06) or younger children (6–11 month vs 30–36 month: coefficient = 0.23 95%CI 0.17, 0.28; 12–17 month vs 30–36 month: coefficient = 0.19 95%CI 0.15,0.24; 18–23 vs 30–36 month: coefficient = 0.09 95%CI 0.04,0.13) had more severe anemia.

**Conclusion:**

The prevalence of moderate-to-severe anemia in these children was about 25%. Province-level region, iron pot use, family size, ethnicity, age and gender of children and severity of maternal anemia were important determinants of the severity of childhood anemia. These findings have some important implications for health policy decision for childhood anemia in rural western China.

## Introduction

Anemia is a global public health problem with major consequences for human health and has affected more than 2 billion people worldwide [1], [2]. Among these affected population, children under 5 years is one of the most vulnerable groups, especially those in the first 2 years of life [3].

Although the etiology of childhood anemia is multi-factorial, iron deficiency is usually the predominant cause [3]. Other causes include infectious diseases, deficiencies of micronutrients, inherited conditions, environmental pollutions and so on [3], [4]. The relative importance of the different causes of anemia varies with the regions of the world [2]. Epidemiological evidence has shown that anemia in children impaired psychomotor development and immune competence, led to poor cognitive and physical development, caused mental retardation, and increased their mortality and morbidity [5]–[10].

In general, the severity of anemia is differentiated by the severity of the reduction in hemoglobin (Hb) level [11]. Severe anemia usually comprises a small proportion of the cases in children but may cause a large proportion of the severe morbidity and mortality [3]. A research on malaria-associated severe anemia in Sub-Saharan Africa showed that children admitted to hospital with severe anemia were more likely to die than those without anemia [12]. A world-wide report also showed that moderate-to-severe anemia increased the risk of mortality in the vulnerable population [2].

The current study assessed the severity of anemia and explored its determinants in children under 36 months old with the aim to provide some evidence for prevention and control of childhood anemia, and to help program developers and project managers determine the priority for programs. Moreover, the study can also supply some insights for the policy-makers to improve the strategies against childhood anemia in prevention and management.

## Methods

### Ethics Statement

We obtained the written informed consent forms from all the caretakers on the behalf of the children participants involved in the study after telling them about the process, purpose and confidentiality of the research. The study was reviewed and approved by the Ethics Committee of Medicine College of Xi'an Jiaotong University.

### Setting and study population

The study used the data from the rural primary health care survey which was conducted across 45 counties of 10 province-level regions (five provinces: Gansu, Guizhou, Jiangxi, Qinghai and Sichuan; four Minority autonomous regions: Inner Mongolia, Guangxi, Ningxia and Xinjiang; one city directly under the central government: Chongqing) from June to August 2005. These 10 province-level regions and 45 counties were not sampled randomly but were directly determined by the Chinese Ministry of Health and UNCEF. A multi-stage probability-proportional-to-size sampling (PPS) method was adopted to determine the sample units of the townships and villages [13]: five townships were sampled from each county and then four villages were selected in each sampled township randomly. In each sampled village, a completely random sampling method was adopted to determine 16 households with children under 36 months old. In each sampled household, only one child was selected randomly and his/her caretaker was interviewed to finish a family questionnaire. But in each sampled village, of the 16 pairs of the children under 36 months and their mothers, whose height and weight were all measured, only 8 pairs were extracted randomly for the Hb measured in the field.

### Data collection

All family primary data in the pre-coded structured family questionnaire were reported by the caretakers of the sampled children. All participants signed the informed consent form and had face-to-face interviews by the field interviewers with the unified family questionnaire involving such concerns as the socio-demographical information, occurrence of common childhood diseases (cold and diarrhea), child care, utilization of maternal care service, and so on. After the interview, the height and weight of the child and his/her mother were measured (height of children: WB-**II** Horizontal Length Measuring Instrument, Beijing Sixth Tractor Factory, Beijing, China; weight: YGZ212 Human Scale, Wuxi Measure Factory, Wuxi, China). If the child and his/her mother had also been determined randomly for the measured Hb, their Hb concentrations were measured in the field by using a portable HemoCue system (HemoCue, Inc., Angelholm, Sweden). The altitude above sea level of the township where the sampled families were located was obtained from Google Earth software (Version 6.2.2, Google Inc, USA).

### Study variables

Severity of childhood anemia was a unique outcome variable of interest in the study. All Hb determinations of children and their mothers living in townships located more than 1000 m above sea level were corrected for by Dallman method [14]. Cutoff value to diagnose anemia in children aged 6–36 months was a Hb level <11 g/dl [3]. Due to the lack of the diagnostic criteria of anemia for children under 6 months, the same cutoff value, which is accepted in clinical practice [15]. was adopted for them. Maternal anemia was diagnosed with the cutoff of Hb <12 g/dl [3]. The Hb higher than cutoff value was identified as normal. Further, childhood and maternal anemia was classified into mild anemia (children: Hb 10–11 g/dl; mothers: Hb 10–12 g/dl), moderate anemia (Hb 8–9.9 g/dl) and severe anemia (Hb <8 g/dl). Z score system was used to compute age-specific height Z score (HAZ), age-specific weight Z score (WAZ) and weight-specific height Z score (WHZ) with the height and weight of children, which could assess the nutritional status of the children [16]. Their extreme data were excluded (HAZ below −6 or above +6, WAZ below −6 or above +5, and WHZ below −5 or above +5) [16]. The children with HAZ, WAZ or WHZ less than −2 were identified as stunting, underweight or wasting respectively. The Demographic and Health Survey wealth index involving 5 variables (water supply, type of vehicle, type of television, income resource and type of salts) was used to assess the socioeconomic status (SES) of the household [17]. According to quintiles of the first principal component of the index, SES was classified into poorest, poorer, medium, richer and richest [17].

### Data analysis

The data in the questionnaires was entered into Epidata 3.1 by double entry and SPSS version17 (SPSS Inc, Chicago, IL, USA) was used to make data analysis. The level of statistical significance of analysis was set at 0.05. Taking into account the attenuation characteristic in the distribution of severity of childhood anemia and the possible correlation of the severity of childhood anemia in the same village, the generalized estimated equation (GEE) linear model with a log-gamma link function was used to identify the predictors of the severity of anemia (1 for normal; 2 for mild anemia; 3 for medium anemia; and 4 for severe anemia) while controlling for possible correlation in the severity of anemia among the same village. All possible study variables were together entered into this model by two age groups (<6 months and beyond 5 months). The regression coefficient reflected the direction and severity of anemia.

## Results

### Sample selection and characteristics

Due to different regional location from other province-level areas, Jiangxi was excluded from the study. In total, we collected 12545 family questionnaires available in the survey and obtained the measured Hb of 6711 children and 5340 mothers from 40 counties of the rest 9 province-level areas. The 5834 family questionnaires without the data of childhood hemoglobin were excluded in the study.

The children in the study were living in 487 villages of 196 townships. Table 1 shows the sample characteristics among these children in rural western China. Of the children, over one half were boys, most were cared for mainly by their mothers, more than four-fifths were delivered at township level or above medical sectors and mostly by natural delivery, and more than two-thirds had swallowed oral vitamin A in the previous year. Slightly less than 40% of them were being breastfed and more than a half had been breastfed ever. In these children, the prevalence of diarrhea in the previous two weeks was mild but that of cold was moderate. Though the prevalence of wasting and underweight was much lower than 10%, the prevalence of stunting was approximately 15%. The majority of their mothers and fathers received only primary education and over a half were of Han ethnicity. Less than a half of the mothers had developed an anemia at the time of the survey.

**Table 1:** Family information and sample characteristics among children under 36 months in rural western China.

	N		%
**Family factors**			
SES			
Poorest	1243		18.52
Poorer	1333		19.86
Medium	1174		17.49
Richer	1433		21.35
Richest	1528		22.78
Family size (≥5)	3457		51.51
Child Size (only one)	3947		58.81
Iron pot use	6326		94.26
Cooking alone for the child	3549		52.88
Mother care	5321		79.29
Maternal age (year) Mean(SD) [range]	27.1(4.8) [15.7–51.7]		
Maternal education(>9years)	594		8.90
Father education(>9 years)	870		13.02
Han ethnicity	3940		58.71
Maternal anemic status^a^			
Normal	3069		57.47
Mild anemia	1739		32.57
Moderate anemia	475		8.90
Severe anemia	57		1.07
**Childhood factors**			
Boy	3703		55.18
Age of children			
0–5 month	832	12.40	
6–11 month	1562	23.28	
12–17 month	1232	18.36	
18–23 month	1172	17.46	
24–29 month	898	13.38	
30–36 month	1015	15.12	
Delivery at township level or above hospitals	5468	81.48	
Natural delivery	5590	83.30	
Vitamin A in the previous year^b^	4553	69.85	
Catching diseases in the previous 2 weeks			
Cold	1262	18.80	
Diarrhea	551	8.21	
Nutritional status*^c^*			
Stunting	989	14.88	
Wasting	372	5.59	
Underweight	542	8.10	
Being breastfed	2680	39.93	
Breastfed ever but not now	3733	55.63	

a 1371 cases were missing; *^b^*193 cases were missing; *^c^*64, 59 and 18 cases in stunting, wasting and underweight were missing respectively.

SD: Standard error; SES: socio-economical status.

### Severity of childhood anemia

**Figure 1:**
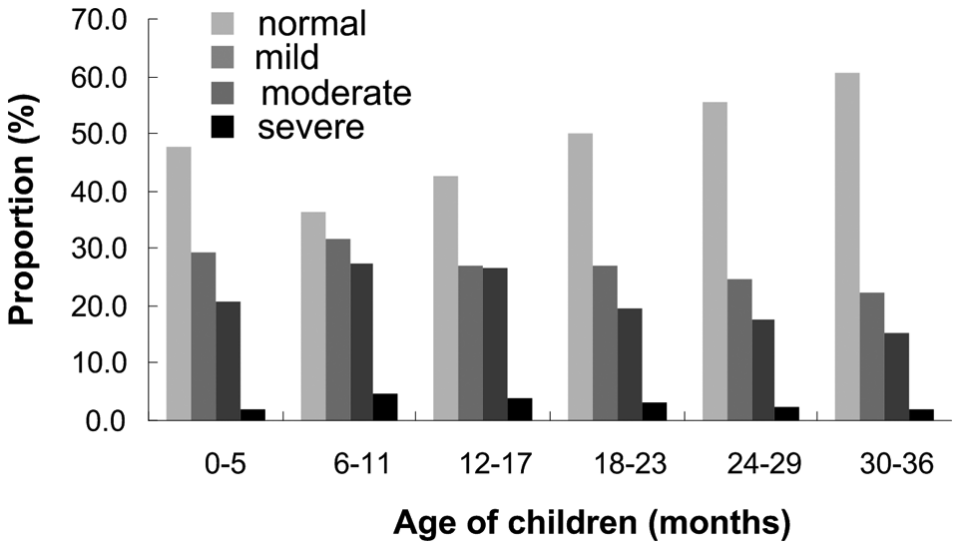
Severity of anemia in six age groups among children under 36 **months in rural western China.**

Table 2 depicts the severity of anemia in children under 36 months in 9 province-level regions of rural western China. The prevalence of anemia among children younger than 36 months in rural western China was 52.5% (95%CI 51.3%–53.7%), of which mild anemia covered 27.4%, moderate anemia 21.9% and severe anemia 3.2%. Among the 9 province-level regions, Qinghai had the highest prevalence of anemia and Inner Mongolia the lowest. The analysis of the severity of childhood anemia shows that the prevalence of moderate and severe anemia was the highest but that of mild anemia the lowest in Qinghai, that of mild anemia the highest in Guizhou, and that of moderate and severe anemia the lowest in Sichuan. Figure 1 describes severity of childhood anemia in six month-age groups. Among six age groups, the prevalence of 3 types of anemia in children aged 6–11 months were the highest and the prevalence of mild and moderate anemia in those aged 30–36 months ranked the lowest. In addition, the prevalence of severe anemia was higher than 3% in 3 age groups –6–11 months, 12–17 months and 18–23 months but was only 1.9% in children aged 0–5 months. In every type of anemia, from the 6–11 month-age group on, the gradually decreasing tendency could be observed clearly.

**Table 2:** Severity of anemia among children 36 months old in rural western China.

Province-level regions	Normal	Childhood anemia			
		All	Mild	Moderate	Severe
Gansu	166(52.87)	148(47.13)	94(29.94)	49(15.61)	5(1.59)
Guangxi	429(54.24)	362(45.76)	214(27.05)	138(17.45)	10(1.26)
Guizhou	297(46.33)	344(53.67)	220(34.32)	117(18.25)	7(1.09)
Inner Mongolia	370(58.27)	265(41.73)	163(25.67)	95(14.96)	7(1.10)
Ningxia	351(55.54)	281(44.46)	167(26.42)	108(17.09)	6(0.95)
Qinghai	260(27.25)	694(72.75)	214(22.43)	378(39.62)	102(10.69)
Sichuan	485(56.66)	371(43.34)	245(28.62)	125(14.60)	1(0.12)
Xinjiang	474(42.21)	649(57.79)	312(27.78)	272(24.22)	65(5.79)
Chongqing	358(46.80)	407(53.20)	209(27.32)	187(24.44)	11(1.44)
All	3190(47.53)	3521(52.47)	1838(27.39)	1469(21.88)	214(3.19)

### Determinants of severity of childhood anemia

Table 3 lists the predictors of severity of anemia among children under 36 months old in rural western China. GEE model analysis shows that province-level region and severity of maternal anemia affected severity of childhood anemia not only in 0–5 months but also beyond 6 months. In addition, for children aged 0–5 months, those in families using iron pot (coefficient = −0.26 95%CI −0.41,−0.12) had seldom more severe anemia. For children aged 6–36 months, those in families with more than 4 members (coefficient = −0.03 95%CI −0.06,−0.01) or of Han ethnicity (coefficient = −0.08 95%CI −0.13,−0.04) seldom developed more severe anemia; boys (coefficient = 0.03 95%CI 0.01,0.06) had more severe anemia than girls; age of children (6–11 month vs 30–36 month: coefficient = 0.23 95%CI 0.17, 0.28; 12–17 month vs 30–36 month: coefficient = 0.19 95%CI 0.15,0.24; 18–23 vs 30–36 month: coefficient = 0.09 95%CI 0.04,0.13) was negatively associated with more severe anemia before 24 months.

**Table 3:** Predictors of severity of anemia among children under 36 months old in rural western China.

Predictors	<6 months of age		6–36 months of age	
	Univariate	Multivariate	Univariate	Multivariate
	Coefficient (95%CI)	Coefficient (95%CI)	Coefficient (95%CI)	Coefficient (95%CI)
**Province-level regions**				
Gansu	−0.21(−0.38, −0.04)	−0.11 (−0.30,0.08)	−0.07(−0.19,0.05)	−0.06 (−0.19,0.07)
Guangxi	−0.11(−0.27,0.04)	−0.15 (−0.33,0.04)	−0.08(−0.19,0.02)	−0.18 (−0.29, −0.06)
Guizhou	−0.15(−0.31,0.01)	−0.24 (−0.43, −0.05)	−0.02(−0.11,0.08)	−0.12 (−0.22, −0.03)
Inner Mongolia	−0.16(−0.31, −0.01)	−0.11 (−0.29,0.08)	−0.12(−0.21, −0.02)	−0.14 (−0.24, −0.05)
Ningxia	−0.18(−0.33, −0.02)	−0.10 (−0.29,0.08)	−0.09(−0.18,0.01)	−0.10 (−0.20, −0.01)
Qinghai	0.04(−0.12,0.20)	0.06 (−0.12,0.24)	0.27(0.18,0.37)	0.16 (0.06,0.25)
Sichuan	−0.26(−0.41, −0.12)	−0.26 (−0.41, −0.11)	−0.11(−0.21, −0.02)	−0.16 (−0.25, −0.07)
Xinjiang	−0.04(−0.18,0.10)	−0.17 (−0.35,0.00)	0.08(−0.01,0.18)	−0.04 (−0.14,0.06)
Chongqing	0	0	0	0.
**Family size with more than 4 members**	−0.03(−0.09,0.04)	−0.01 (−0.08,0.06)	−0.02(−0.04,0.01)	−0.03 (−0.06, −0.01)
**Iron pot use**	−0.25(−0.37, −0.12)	−0.26 (−0.41, −0.12)	−0.05(−0.11,0.00)	−0.06 (−0.12,0.01)
**Han ethnicity**	−0.09(−0.16, −0.02)	−0.05 (−0.16,0.05)	−0.10(−0.14, −0.06)	−0.08 (−0.13, −0.04)
**Boy**	0.02(−0.05,0.09)	0.04 (−0.04,0.11)	0.05(0.03,0.07)	0.03 (0.01,0.06)
**Age of children**				
6–11 mo	-	-	0.23(0.20,0.27)	0.23 (0.17,0.28)
12–17 mo	-	-	0.21(0.17,0.25)	0.19 (0.15,0.24)
18–23 mo	-	-	0.12(0.08,0.16)	0.09 (0.04,0.13)
24–29 mo	-	-	0.05(0.01,0.10)	0.02 (−0.02,0.07)
30–36 mo	-	-	0	. 0
**Maternal anemic status**				
Severe anemia	0.18(−0.11,0.46)	0.15(−0.13,0.42)	0.21(0.06,0.37)	0.20 (0.04,0.36)
Moderate anemia	0.30(0.18,0.42)	0.31 (0.19,0.43)	0.18(0.12,0.25)	0.17 (0.11,0.24)
Mild anemia	0.10(0.03,0.17)	0.14 (0.06,0.22)	0.09(0.06,0.12)	0.09 (0.06,0.12)
Normal		0	0	0.

Only predictors at 5% of multivariate GEE model were listed.

## Discussion

In rural western China, the nutritional status of infants and young children was poor [18], [19]. Nutritional anemia in children, as a result of nutritional deficiencies, also became a prominent problem. Our study found that the prevalence of anemia among the children younger than 36 months was 52.5% which was significantly lower than 72.6% in Burma [4] and slightly lower than 55.3% in Bangladesh [20]. In spite of this, according to the WHO's classification standard of anemia as a problem of public health significance [11], the prevalence of anemia among the children in rural western China had become a severe public health problem. So a nation-level comprehensive prevention and control strategy of childhood anemia is urgently needed to strengthen the prevention and control of childhood anemia among the children in rural western China. Certainly, regional imbalance in the prevalence of childhood anemia also became quite obvious. In Qinghai, the prevalence of childhood anemia was higher than 70%, but that of Inner Mongolia was only slightly higher than 40%. So, prevention and control programs of childhood anemia should address the geographical difference in prevalence of childhood anemia and national effort should also encourage local health authorities to draw up regional strategies for childhood anemia based on the analysis of local survey of childhood anemia.

In addition, the prevalence of severe anemia among children had surpassed 3%. However, where severe anemia was common (2% or more prevalence of a population group), its detection and treatment in primary care facilities was necessary to prevent morbidity and mortality from severe anemia [3]. The prevalence of severe anemia in Qinghai and Xinjiang was much higher than that in other province-level regions. GEE model analysis also showed that regional effect on the severity of childhood anemia was significantly great, especially in children aged 6–36 months. More importantly, those areas with the most widespread and severe anemia often had the most limited resources [3]. It is important to prioritize program efforts so that the limited resources can be most effectively used in these regions. Also, poor health care services in these areas may restrict the implementation of some effective therapeutic measures for severe anemia. The strength of the basic health service systems for childhood anemia was also a problem to be solved. It was also important that primary health care providers be able to recognize these cases and treat or refer to individuals with severe anemia [3]. Moreover, it was worth considering that severe anemia may have more complicated etiology mechanism. In the province-level regions with much higher prevalence of severe anemia, surveys should be more inclusive and collect information on iron status and other causes of anemia [1]. For children with severe anemia which is unresponsive to iron therapy, other etiological factors need to be detected and appropriate treatment should also be taken to reduce the mortality and morbidity due to anemia.

GEE model analysis showed that the severity of maternal anemia was an important factor influencing the severity of childhood anemia not only in children under 6 months but also in those beyond 6 months. Breast milk was a main nutritional resource for most of the children under 24 months, especially for those under 6 months. More severe maternal anemia may reduce more iron content of breast milk [21], [22]. Additionally, mothers and their children shared a sociological environment and within 12 months after birth, their dietary quality may be similar [21]. Thus improving the severity of maternal anemia during the breastfeeding period was crucial to reduce the occurrence of anemia and decrease its severity among breastfed children.

Our study also found that in children aged 6–36 months those in the family with more than 4 members seldom developed more severe anemia. Plausibly, multi-member families seemed to be equipped with a higher and better ability of caring for young children than those with fewer members. As a result, the diet and health status of young children could be taken good care of by particular members. Moreover, multi-member families were usually in good economic situation, which could make richly nutritious food available for their children. In the children aged 6–36 months, those of Han ethnicity also seldom developed more severe anemia than those of minority. A previous study of the dietary intake of 12 minority ethnicities in China had showed that in the dietary structure of minority residents, the dietary imbalance problem exists to a certain extent [23]. The dietary imbalance may easily cause a low intake of some important nutrients such as iron, vitamin B12 and so on. So, nutrition education programs should guide the minority families to utilize the local food resources scientifically and advocate the principle of a balanced diet for their young children so as to reduce the risk of moderate-to-severe anemia in their young children.

Moreover, our study also found that age of a child was associated negatively with more severe anemia in children aged 6–24 months. The similar effect of the age of the child on anemia has been observed in the previous studies in rural India and Burma [4], [21]. Perhaps childhood physiology was predominant. Younger children need a relatively higher iron intake to meet the requirement of rapid growth. For children only just beyond 6 months of age, the iron in breast milk is not much enough. Also, most standard diets do not supply enough iron for children aged 6–24 months [14]. So, supplementary rich-iron foods need to be introduced to children at six months, when maternal iron stores are exhausted [14]. Additionally, some infectious diseases became susceptible to children beyond 6 months. So preventing and treating these infections timely is also important for childhood anemia in this age. In the 3 age groups (i.e 6–11 mo, 12–17 month and 18–23 month) the prevalence of severe anemia was higher than 3%. So, more attentions should be paid to children aged 6–23 months.

Although the effect of the gender on childhood anemia was controversial [24], our findings that boys were likely to suffer from more severe anemia than girls was well supported by a similar study in rural India [21]. A possible reason may be that there is a greater absolute longitudinal growth among boys than girls [21]. Certainly, a recent study by Lin et al in children and adolescents with intellectual disabilities showed that girls were more inclined to be anemic than boys with intellectual disability [25]. Some other studies showed there was no effect of the gender on childhood anemia [24], [26]. So further study should be conducted to confirm this phenomena observed in our study.

Our study had several strengths. It was based on a large representative sample and all subjects were recruited from the general population. In addition, the study was to support and extend the findings of the studies on the prevalence of childhood anemia and to highlight some important determinants of the severity of childhood anemia.

Our study had also certain limitations. The information of supplementary feeding was not available in the study, which may affect our predictors of the severity of childhood anemia. Moreover, other limitations such as potential biases, unobserved heterogeneity, etc. may exist in the study.

In conclusion, the prevalence of moderate-to-severe anemia among children under 36 months is about 25%. Socio-demographical factors had less influence on the severity of anemia among children younger than 6 months than those aged 6–36 months. Province-level region, iron pot use, family size, ethnicity, age and gender of children and severity of maternal anemia are important determinants of the severity of childhood anemia. These findings have some important implications for health policy decision for childhood anemia in rural western China.
