# Correction: Severity of Anemia among Children under 36 Months Old in Rural Western China

**DOI:** 10.1371/annotation/1182aef6-c07e-4391-919b-cda801ec3f40

**Published:** 2013-07-01

**Authors:** Wenlong Gao, Hong Yan, DuolaoWang, Shaonong Dang, Leilei Pei

The name of the third author was given incorrectly. The correct name is: Duolao Wang. The correct citation is: Gao W, Yan H, Wang D, Dang S, Pei L (2013) Severity of Anemia among Children under 36 Months Old in Rural Western China. PLoS ONE 8(4): e62883. doi:10.1371/journal.pone.0062883.

In addition, the first sentence in Contributions was incorrectly separated into two sentences. The correct sentence is: "Conceived and designed the experiments: WG SD LP." 

