# Correction: Severity of Anemia among Children under 36 Months Old in Rural Western China

**DOI:** 10.1371/annotation/f03fa448-2129-414a-b841-031ea683d965

**Published:** 2013-09-13

**Authors:** Wenlong Gao, Hong Yan, DuolaoWang, Shaonong Dang, Leilei Pei

There was an error in the Author Contributions Statement. The Author Contributions Statement should read: "Conceived and designed the experiments: WG SD LP. Performed the experiments: WG HY SD. Analyzed the data: WG DW. Contributed reagents/materials/analysis tools: WG HY. Wrote the paper: WG HY." 

